# Fracture Strength of Monolithic Zirconia Crowns with Modified Vertical Preparation: A Comparative *In Vitro* Study

**DOI:** 10.1055/s-0041-1735427

**Published:** 2021-11-30

**Authors:** Marwah Ismael Abdulazeez, Manhal A. Majeed

**Affiliations:** 1 Department of Restorative and Esthetic Dentistry, College of Dentistry, Baghdad University, Baghdad, Iraq

**Keywords:** zirconia, vertical, reverse shoulder

## Abstract

**Objective** The aim of this study was to evaluate the influence of different marginal designs (deep chamfer, vertical, and modified vertical with reverse shoulder) on the fracture strength and failure modes of monolithic zirconia crowns.

**Materials and Methods** Thirty sound human maxillary first premolar teeth with comparable size were used in this study. The teeth were divided randomly into three groups according to the preparation design ( *n* = 10): (1) group A: teeth prepared with a deep chamfer finish line; (2) group B: teeth prepared with vertical preparation; and (3) group C: teeth prepared with modified vertical preparation, where a reverse shoulder of 1 mm was placed on the buccal surface at the junction of middle and occlusal thirds. All samples were scanned by using an intraoral scanner (CEREC Omnicam, Sirona, Germany), and then the crowns were designed by using Sirona InLab 20.0 software and milled with a 5-axis machine. Each crown was then cemented on its respective tooth with self-adhesive resin cement by using a custom-made cementation device. A single load to failure test was used to assess the fracture load of each crown by using a computerized universal testing machine that automatically recorded the fracture load of each sample in Newton (N).

**Statistical Analysis** The data were analyzed statistically by using one-way analysis of variance test and Bonferroni test at a level of significance of 0.05.

**Results** The highest mean of fracture load was recorded by chamfer (2,969.8 N), which followed by modified vertical (2,899.3 N) and the lowest mean of fracture load was recorded by vertical (2,717.9 N). One-way ANOVA test revealed a significant difference among the three groups. Bonferroni test showed a significant difference between group A and group B, while a nonsignificant difference was revealed between group C with group A and group B.

**Conclusion** Within the limitations of this in vitro study, the mean values of fracture strength of monolithic zirconia crowns of all groups were higher than the maximum occlusal forces in the premolar region. The modification of the vertical preparation with a reverse shoulder placed at the buccal surface improved the fracture strength up to the point that it was statistically nonsignificant with the chamfer group.

## Introduction

Zirconia has gained popularity because of its superior mechanical properties related to the transformation toughening mechanism. 1 Full-contour zirconia restorations can be used successfully omitting the veneering porcelain layer, which is more vulnerable to chipping. 2 Currently, monolithic zirconia can be milled even with a reduced thickness due to its high flexural strength, which affords adequate strength even for the posterior fixed dental prosthesis. 3 4 5

Over the years, the horizontal preparation using the chamfer and shoulder finish lines has been accepted as the gold standard for all-ceramic restoration. However, these types of margins are invasive in terms of sound tooth structure removal that is critical for biological and esthetic concepts. 6 The introduction of high-strength polycrystalline materials allowing the use of vertical preparation as a less extensive alternative to the horizontal (chamfer and shoulder). 7 8 9 The vertical margins can provide the most acute marginal restoration that preserves maximum sound tooth structure 10 ; this is crucial for vital teeth and root canal-filled teeth to reduce stresses on the abutment tooth when restored with a crown. 11 Nevertheless, the type of the restoration margin appears to be the most technically challenging issue as cracks may be induced from the occlusal surface to the thin margin. 12

In this study, the vertical preparation was modified with a reverse shoulder at the buccal surface of the abutment tooth. This approach has been adopted from a group of Italian clinicians (Tomorrow Tooth Group), who claim that this approach improves the esthetics and biomechanics of zirconia crowns with vertical preparation. 6 However, no scientific articles are available regarding this approach.

## Materials and Methods

Thirty sound human maxillary first premolar teeth extracted for orthodontic treatment within the range of age from (18–22) years were collected to be used in this study. The selected teeth should have a comparable size that was checked with the digital vernier and should have no caries, restorations, or cracks as examined under a digital microscope (Dino-Lite capture 2.0, version 1.3.6., Taiwan) at a magnification of (×40). The teeth were stored in thymol solution for 1 week at room temperature to avoid fungal and bacterial infection and then settled in distilled water to prevent dehydration.

Each tooth sample was embedded 2 mm below the cemento-enamel junction (CEJ) in a specially fabricated square rubber mold containing freshly mixed cold cure acrylic, a modified dental surveyor (Paraline, Dentaurum, Germany) was used for alignment of the long axis of the tooth to be vertical to the horizontal plane of the mold.

Samples were divided randomly into three groups ( *n* = 10) according to the preparation design: Group A: teeth prepared with a horizontal preparation (chamfer finish line); Group B: teeth prepared with vertical preparation; Group C: teeth prepared with modified vertical preparation (reverse shoulder).

All samples were prepared using a dental surveyor for standardization purposes. The vertical arm of the surveyor was modified to grasp a high-speed turbine (W&H, Austria) to ensure the parallelism between the long axis of the bur and the long axis of the tooth that was checked with a protracter.

**Fig. 1:**
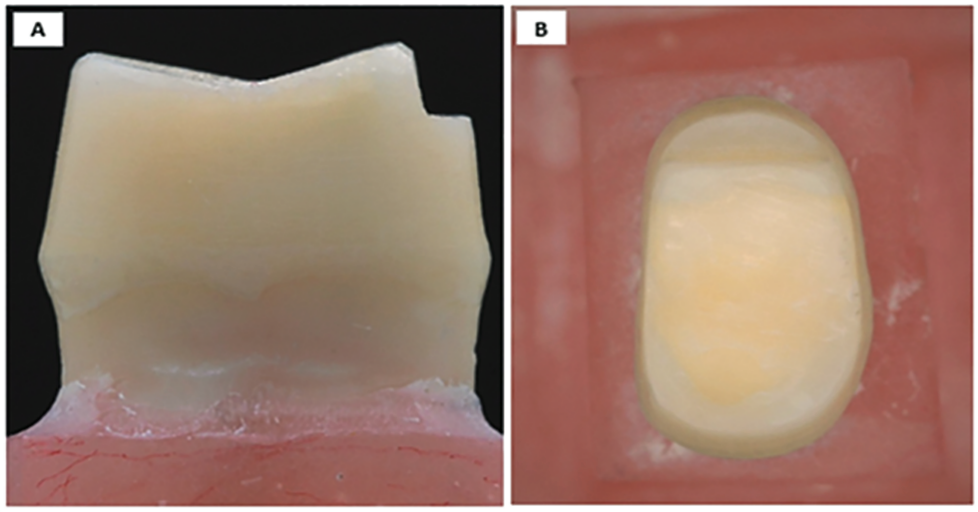
Modified vertical preparation with reverse shoulder. ( **A** ) Proximal view and ( **B** ) occlusal view.

All teeth received a standardized tooth preparation with a 4 mm axial height that was measured from the mesial surface to the finish line placed 1 mm above the CEJ. For the teeth in group A, the chamfer margin design of 0.8 mm depth was prepared by using a round-end tapered fissure diamond bur (6856 314 016, Komet, Germany) with a total convergence angle of 6 degrees; for the teeth in group B&C vertical margin design was prepared using a round safe end tapered diamond bur (851 314 012, Komet, Germany) with a total convergence angle of 4 degrees; for group C, the reverse shoulder of 1 mm depth was prepared on the buccal surface 1.5 mm from the occlusal surface by using a flat-end diamond fissure bur with guide-pin (8372P 314 023, Komet, Germany). All these measurements were checked with a digital vernier ( Fig. 1 ).

A digital impression was then captured for each tooth by using CEREC Omnicam intraoral scanner (Sirona, Germany). The crowns were then designed by using Sirona InLab CAD 20.0 software and milled out of zirconia blanks (IPS e.max ZirCAD LT; Ivoclar Digital, Germany) with a 5-axis milling unit (In-Laboratory MC ×5 milling machine, Sirona, Germany). The milled crowns were then subjected to the sintering process to obtain the original size, strength, and color by using In Fire HTC Speed Sintering Furnace (Sirona, Germany) at 1,450°C.

The crowns were then glazed with glaze paste (FLUO Ivocolor glaze paste; Ivoclar Vivadent, Liechtenstein) by using a brush. Crystallization/glaze firing was done by using the Programat P500 furnace (Ivoclar, Germany) at 840°C for 20 minutes. The intaglio surface of each crown was then sandblasted with aluminum oxide particles ≤50 μm and 1 bar at a distance of 10 mm for 15 seconds according to the manufacturers’ instructions to increase the mechanical interlocking between zirconia and the luting cement by using a sandblasting machine (Renfert, Germany).

Each crown was then cemented on its respective tooth by using a self-adhesive resin cement (RelyX U200 3M ESPE, Germany). The crown was initially seated by finger pressure, and then a vertical load of 5 kg was applied for 6 minutes by using a custom-made cementation device. All samples were then stored in distilled water at 37°C for 24 hours.

The fracture loads of the crowns were measured with a single load to failure test by an electronic-controlled universal testing machine (Laryee, China). A round-end stainless steel indenter at a crosshead speed of 0.5 mm/min was used to apply a vertical load on each zirconia crown. To avoid distortion through direct contact between the indenter and the crown, a piece of thin rubber (1 mm thickness) was placed between the indenter and the crown. All crowns were loaded until a failure occurs, and the fracture load was recorded automatically in Newton (N).

A digital microscope (Dino-Lite capture 2.0, version 1.3.6., Taiwan) was then used for assessment of the modes of fracture for all samples at a magnification of ×70 according to Burke’s classification 13 ( Table 1 ).

**Table 1:** Burke’s classification for the mode of fracture

Mode of fracture	Description
Code I	Minimal fracture or crack in the crown
Code II	Less than half of the crown lost
Code III	Crown fracture through midline (half of the crown displaced or lost)
Code IV	More than half of the crown lost
Code V	Severe fracture of the crown and/or tooth

Statistical analysis was done by using Statistical Package for Social Science (SPSS version 16); the normal distribution of variables was evaluated with Shapiro–Wilk test; one way ANOVA test was used to look for the significance of the mean difference of fracture strength among the three groups; and Bonferroni correction was used for multiple comparisons among the groups.

## Results

Shapiro–Wilk test revealed that the data were normally distributed ( *p* > 0.05). The descriptive statistics including the mean, standard deviation, and minimum and maximum values of the fracture strength in Newton (N) for the three groups are shown in Table 2 .

**Table 2:** Data for the fracture strength (mean and standard deviation) in Newton

Group	Mean	SD
Deep chamfer	2,969.80	182.989
Vertical	2,717.90	241.774
Modified vertical	2,899.30	164.868
Abbreviation: SD, standard deviation.		

The highest mean value of the fracture strength was recorded by group A (2,969.8 ± 182.9 N), in which the crowns were prepared with a chamfer finish line, followed by group C (2,899.3 ± 164.8) and the lowest mean value was recorded by group B (2,717.9 ± 241.7 N), in which the crowns were prepared with vertical preparation.

One-way ANOVA test was used for comparison of the fracture strength among the three groups at a level of significance of 0.05 and revealed a statistically significant difference among the groups ( *p* < 0.05; Table 3 ). Bonferroni test showed that there were statistically significant differences in the values of fracture strength between groups A and B ( *p* < 0.05). On the other hand, no statistically significant difference was found neither between group A and C nor between group B and C ( *p* > 0.05; Table 4 ).

**Table 3:** One-way ANOVA for comparison of fracture strength among the three groups

ANOVA	Sum of squares	Df	Mean square	F	Sig.
Between groups	337,766.067	2	168,883.033	4.253	0.025
Within groups	1,072,086.600	27	39,706.911		
Total	1,409,852.667	29			
Abbreviations: ANOVA, analysis of variance; Sig., significance.					

**Table 4:** Bonferroni test for multiple comparisons of fracture strength of three groups between each other

Group (I)	Group (J)	Mean difference (I–J)	Standard error	Significance
A	B	251.900	89.114	0.026
	C	70.500	89.114	1.000
B	C	−181.400	89.114	0.155

Concerning the modes of fracture in this study, the majority of samples with chamfer finish line (90%) showed a severe fracture of the tooth and/or restorations (code V). Whereas 60% of samples with vertical and modified vertical preparation showed code V and (40%) showed a fracture of the restoration only (code II, III, and IV; Table 5 ).

**Table 5:** Modes of fracture of three groups

Group	Code I (%)	Code II (%)	Code II (%)	Code IV (%)	Code V (%)	Total (%)
A				1 (10%)	9 (90%)	10 (100%)
B		1 (10%)	1 (10%)	2 (10%)	6 (60%)	10 (100%)
C			2 (20%)	2 (20%)	6 (60%)	10 (100%)

## Discussion

Tooth preparation design is a specially vital factor in determining the strength of all-ceramic crowns. 14 A reduction of dental hard tissue is essential with full coverage restorations to secure structural durability and restore natural anatomy and esthetics. 15 The high mechanical properties of zirconia enable the fabrication of monolithic zirconia crowns with minimal invasive vertical preparation. 16

The results of this study revealed that margin design has an effect on the fracture load of the crown restoration. This is consistent with other in vitro studies. 5 17 18 While other studies found no such effects. 16 19 20 However, these conflicting results could be due to the different experimental settings for fracture load assessment as well as the tested materials.

The chamfer margin design showed a significantly higher fracture load than the vertical margin design; this could be attributed to the increased thickness of the restoration that carries the occlusal forces, and this results in less stress concentration on axial walls of the substrate. 21 Also, it has been found that increasing the crown margin thickness leads to fracture at a higher load. 18 22

It is noteworthy that no previous studies are available in the literature concerning the modification of the vertical preparation with a reverse shoulder. However, interestingly, the fracture loads obtained with the modified vertical preparation were higher than the vertical group; this could be related to that the reverse shoulder allows more thickness of material on the axial wall, which results in a more favorable stress distribution.

Remarkably, despite the minimal thickness of the crown margins with vertical preparation groups, the mean values of fracture strength of crowns were higher than the maximum mastication forces in the premolar region (900 N). 23 This could be due to the superior mechanical properties of zirconia that is advantageous for minimal preparation design. This showed agreement with the result by other in vitro studies, suggesting that monolithic zirconia crowns can be advocated with a reduced thickness in the posterior region. 3 5 24 Moreover, a clinical study revealed that the performance of full-contour zirconia crowns with feather edge and chamfer finish lines showed no differences in survival and success rates after 4 years of clinical service. 25

The findings of this study are inconsistent with Reich et al 17 and Beuer et al 26 ; they concluded that zirconia coping with knife edge preparations had higher fracture loads related to those with the chamfer preparations. However, they used zirconia copings while monolithic zirconia crowns were tested in the current study.

Similar findings by Mitov et al 2 and Jasim et al 5 who attained higher fracture strength with shoulderless preparation compared with the chamfer, they tested monolithic zirconia crowns cemented on metal dies that have higher elastic modulus than dentin, and this might increase the fracture strength values. 27 However, the fracture resistance of the restorations could give more precise results if natural teeth were used as abutments. 28

On the other hand, Kasem et al 16 demonstrated a nonsignificant difference between the fracture loads of monolithic translucent zirconia crowns cemented on natural teeth with vertical and horizontal preparations.

The results of the present study are concurrent with Findakly and Jasim 29 who reported lower fracture resistance of monolithic zirconia crowns with vertical margins compared with the horizontal (shoulder) margins, they used the same fracture test, cement, and material in this study (IPS e.max ZirCAD LT, Ivoclar Vivadent). The fracture values obtained for the vertical (2,300 N) were lower than values obtained in the current study (2,717 N). This could be attributed to other variables like thermocycling as several studies found that the flexural strength of zirconia was reduced with thermocycling. 30 31 32

Regarding modes of failure, 90% of samples with deep chamfer margins showed a severe fracture of tooth and restoration (catastrophic failure), whereas 60% of samples with vertical and modified vertical showed this mode of failure; this could be attributed to the increased depth of preparation with the horizontal finish line that leads to a decrease in the fracture resistance of the tooth. 33 For all groups, more than half of the samples showed catastrophic failure. This could be related to the high strength of low translucent zirconia used in this study (IPS e.max ZirCAD LT, Ivoclar Vivadent) that is classified as 3 yttria-stabilized tetragonal zirconia polycrystal (3 Y-TZP) with no cubic phase. Increasing the cubic phase with high translucent zirconia (4Y-TZP) results in limitation of the transformation toughening mechanism (tetragonal to monoclinic phase transformation) and consequently reduces the mechanical properties. 34 35

One of the limitations of this in vitro study is that the crowns were subjected to static load failure test without artificial aging processes, such as thermocycling and cyclic loading that could provide more information about the clinical performance of the restoration. However, a single load to failure test is still important and considered as the cornerstone for testing materials as a first step.

## Conclusion

Within the limitations of this in vitro study, the following conclusions could be drawn:

- The mean values of fracture strength of monolithic zirconia crowns in all groups were higher than the maximum biting forces in the premolar area.
- Modified vertical preparation with reverse shoulder improved the fracture strength of monolithic zirconia crowns up to the point that it becomes nonsignificant with chamfer preparation.
